# Outdoor Treatment of Consumption

**Published:** 1898-04-16

**Authors:** 


### OUTDOOR TREATMENT OF CONSUMPTION.

Dr. Francis Potts, of Bournemouth, writes to us in regard to recent articles in the The Hospital on the open-air treatment of phthisis, drawing our attention to a house which he has had fitted up at Bournemouth for carrying out this mode of treatment. The house is managed under the direct control of Dr. Potts, and the photographs which he has sent to us, together with his description of the precautions takenare very attractive.

